# Identification of Conomarphin Variants in the *Conus eburneus* Venom and the Effect of Sequence and PTM Variations on Conomarphin Conformations

**DOI:** 10.3390/md18100503

**Published:** 2020-10-01

**Authors:** Corazon Ericka Mae M. Itang, Jokent T. Gaza, Dan Jethro M. Masacupan, Dessa Camille R. Batoctoy, Yu-Ju Chen, Ricky B. Nellas, Eizadora T. Yu

**Affiliations:** 1 Institute of Chemistry, College of Science, University of the Philippines, Diliman, Quezon City 1101, Philippines; erickaitang@gmail.com (C.E.M.M.I.); jtgaza@up.edu.ph (J.T.G.); drbatoctoy@up.edu.ph (D.C.R.B.); rbnellas@up.edu.ph (R.B.N.); 2 Marine Science Institute, College of Science, University of the Philippines, Diliman, Quezon City 1101, Philippines; djmasacupan@gmail.com; 3 Institute of Chemistry, Academia Sinica, No. 128, Section 2, Academia Rd, Nankang, Taipei 115, Taiwan; yujuchen@gate.sinica.edu.tw

**Keywords:** conopeptide, proteomics, 3D structure, conomarphins

## Abstract

Marine cone snails belonging to the Conidae family make use of neuroactive peptides in their venom to capture prey. Here we report the proteome profile of the venom duct of *Conus eburneus*, a cone snail belonging to the Tesseliconus clade. Through tandem mass spectrometry and database searching against the *C. eburneus* transcriptome and the ConoServer database, we identified 24 unique conopeptide sequences in the venom duct. The majority of these peptides belong to the T and M gene superfamilies and are disulfide-bonded, with cysteine frameworks V, XIV, VI/VII, and III being the most abundant. All seven of the Cys-free peptides are conomarphin variants belonging to the M superfamily that eluted out as dominant peaks in the chromatogram. These conomarphins vary not only in amino acid residues in select positions along the backbone but also have one or more post-translational modifications (PTMs) such as proline hydroxylation, C-term amidation, and γ-carboxylation of glutamic acid. Using molecular dynamics simulations, the conomarphin variants were predicted to predominantly have hairpin-like or elongated structures in acidic pH. These two structures were found to have significant differences in electrostatic properties and the inclusion of PTMs seems to complement this disparity. The presence of polar PTMs (hydroxyproline and γ-carboxyglutamic acid) also appear to stabilize hydrogen bond networks in these conformations. Furthermore, these predicted structures are pH sensitive, becoming more spherical and compact at higher pH. The subtle conformational variations observed here might play an important role in the selection and binding of the peptides to their molecular targets.

## 1. Introduction

The venom of marine cone snails is a goldmine for neuroactive peptides known as conopeptides. Current estimates report that cone snail venom consists of hundreds to thousands of conopeptides that act on a wide range of pharmacological targets such as ion channels and G protein-coupled receptors [1,2,3]. For this reason, these conopeptides are being explored as lead compounds for drug discovery and development [3,4,5,6]. Bioassay-guided fractionation of venom peptides has been very successful in identifying these bioactive peptides, and has paved the way for basic biochemical studies and the elucidation of molecular mechanisms of action of select peptide targets [5,6,7,8,9].

With the advent of high throughput mass spectrometry (MS), proteomic profiling of whole venoms has become possible [10,11,12,13]. Proteomic methods are often integrated with transcriptomic and bioinformatics techniques to obtain a more comprehensive picture of the whole toxin profile of cone snail venoms. This new approach, termed as “integrated venomics”, has revealed that cone snail venoms are highly complex mixtures containing several hundred peptide components [14,15,16]. It provides a comprehensive list of precursor sequences as next-generation sequencing (NGS) tools have the ability to detect cDNA of precursor peptides. Moreover, it allows for the verification of sequences in the mature peptide regions through tandem mass spectrometry to characterize the venom proteins. MS can also detect the presence of covalently-bonded post-translational modifications (PTMs) in mature peptides, thereby confirming that conopeptides are highly diverse [17,18,19]. Global interrogation of whole cone snail venoms have paved the way for obtaining exciting insights into the biochemistry of these conopeptides. MS analysis of milked venom versus venom extracted from venom ducts has revealed differences in protein sequences between the two sets, which point to the influence of sampling or protein extraction methods in the resulting proteomes [20]. Recently, intraspecific variations in conopeptides expressed by different individuals of the same species have also been revealed [21,22,23,24]. Lastly, in addition to intraspecific variations, changes in the venom peptide composition of individuals during feeding events have also been observed through MS studies [25]. There are over 10,000 conopeptides that have now been identified and sequenced via integrated venomics and are compiled in the ConoServer data repository [26,27]. Work is needed to identify more conopeptides and construct a more comprehensive conopeptide library.

Here we report the proteomic profile of the *C. eburneus* venom obtained through high resolution mass spectrometry analysis of crude venom extracts. Furthermore, three-dimensional structures for the dominant conomarphin variants were generated to gain insights on the structural diversity of *C. eburneus* conomarphins, which might play an important role in the selectivity and binding to their molecular targets.

## 2. Results

A total of 711 unique mass components ranging from ~400 Da to ~10,000 Da were identified from the native venom extract of *Conus eburneus*. A unimodal distribution of these mass components was observed, wherein approximately 90.0% of the detected components have masses below 4000 Da. This highlights the short nature of conopeptides and is consistent with previous findings that reported a conopeptide length range from 10 to 45 amino acids (~1 to 5 kDa) [14,18]. Of the 711 masses, 24 conopeptides were identified and sequenced without ambiguity through tandem mass spectrometry (Table 1). The sequences were deduced from the ConoServer database (6275 peptide entries) and the *C. eburneus* transcriptome library (149 transcript entries).

The number of identified conopeptides covers only ~3.38% of the experimentally detected masses. It is likely that the unassigned experimental masses were not matched to peptide sequences due to the presence of PTMs (such as glycosylation) that were not included in the search, or to differential proteolytic cleavage sites during conopeptide maturation, or due to the possible presence of non-peptidic components in the venom. Furthermore, it is also likely that peptides in the crude venom extract were products of protein degradation which may have occurred during venom duct homogenization. It is therefore not unexpected to obtain a higher number of peptide masses compared to the number of toxins made in the venom duct. To further increase the number of sequence matches or assignments of the experimental masses, de novo sequencing algorithms are needed [11,28].

Currently, there are only 27 conopeptides associated with *C. eburneus* in the ConoServer database, of which, only three (δ-ErVIA, conomarphin Eb1, and conomarphin Eb2) have peptide-level evidence [29,30]. These peptides were also identified in this study, in addition to 21 more peptides that were verified by MS/MS (Table 1).

In addition to the 24 peptides in Table 1, we also identified 12 more peptides through stringent mass matching within a ±10ppm mass accuracy (Supplementary Table S1). These peptides are similar to those found in the transcriptome of *C. eburneus* and *C. tessulatus*. Without tandem MS verification, we only suggest that these peptides may also be present in the venom duct of *C. eburneus*.

**Figure 1:**
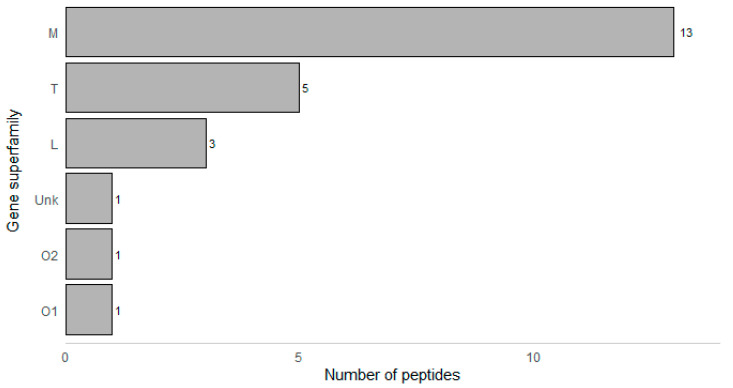
Gene superfamilies of the peptides identified from the proteome of *C. eburneus* venom. Unk—unknown gene superfamily

In terms of conopeptide gene families, 13 of the 24 identified peptides belong to the M gene superfamily while five (5) belong to the T superfamily (M superfamily: 54.2% and T superfamily: 20.8%) (Figure 1). This is consistent with a recent transcriptomics study by Mendoza et al. (2019), which showed higher expression levels of M and T superfamilies, despite having higher diversity of O1 superfamily transcripts [29]. Both the M and T superfamilies are known to be abundant in most cone snail species. The M superfamily is the most diverse, with multiple known pharmacological targets (Na^+^ channels, K^+^ channels, and nAChRs). Meanwhile, conopeptides belonging to the T superfamily are known to target Na^+^ channels, Ca^2+^ channels, noradrenaline transporters, and somatostatin-3 receptors [31].

**Figure 2:**
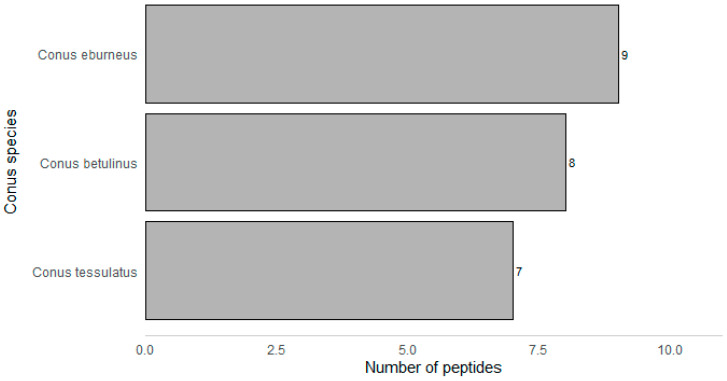
*C. eburneus* peptides that share sequence similarity with *C. betulinus* and *C. tessulatus*.

About a third of the peptides identified in this study were found in the *C. eburneus* transcriptome. Meanwhile, 33.3% share sequence similarity with peptides found in the *C. betulinus* transcriptome and 29.2% with *C. tessulatus* (Figure 2). It is not surprising to find an overlap in the proteome of *C. eburneus* and *C. tessulatus* since they both belong to the same Tesseliconus clade [32]. Hence, they could have evolved a similar set of venom components. In a similar way, *C. betulinus* is also relatively closely related to *C. eburneus* although they do not belong to the same clade [32].

**Figure 3:**
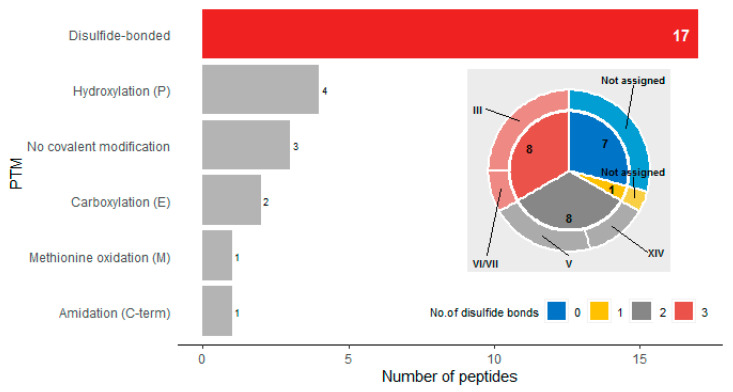
Post-translational modification (PTM) profile of *C. eburneus* proteome. The majority of the venom peptides were found to have disulfide bonds. Inset shows the venom peptides as classified by the cysteine frameworks (outer ring) and the number of peptides with varying degrees of disulfide bonding (inner circle).

The majority of the *C. eburneus* venom peptides were found to have disulfide bonds and can be classified into four major cysteine frameworks—V, XIV, III and VI/VII (Figure 3). Cysteine framework V (-CC-CC-) has been observed for ε-conotoxins that target presynaptic calcium channels while cysteine framework XIV (-C-C-C-C-) has been observed for α-conotoxins that target nicotinic acetylcholine receptors [33]. Both of these frameworks are for peptides with two disulfide bonds. On the other hand, cysteine frameworks III and VI/VII are for peptides with three disulfide bonds. Cysteine framework III (-CC-C-C-CC-) has been observed for ι- and κ-conotoxins that target voltage-gated sodium and potassium channels, respectively, while cysteine framework VI/VII (-C-C-CC-C-C-) has been previously observed in a variety of conotoxins including ω-, λ-, κ-, δ-, and μ-conotoxins [33]. In terms of other covalent post-translational modifications (PTMs), four of the 24 identified peptides were observed to have hydroxyproline residues. It is also interesting to note that we detected several conopeptide variants (unmodified and differentially-modified variants).

**Figure 4:**
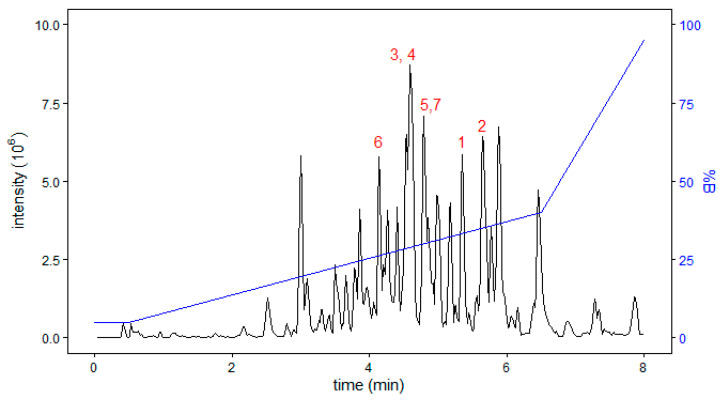
Chromatographic profile of the *C. eburneus* venom duct showing the elution of multiple conomarphin variants. Peaks are labelled with the conomarphin number annotation used in Table 2. The elution gradient is shown as a blue trace.

A striking feature of the *C. eburneus* venom is that all seven of the Cys-free peptides identified in this study are conomarphin variants (Table 2) that eluted out as dominant peaks in the chromatogram (Figure 4). These conomarphins share similar sequences with predicted *C. betulinus* conomarphins Bt1, Bt2, and Bt3 [26,27,34]. Most of the differences in amino acid residues and post-translational modifications lie in positions 7–10 (Table 2).

**Figure 5:**
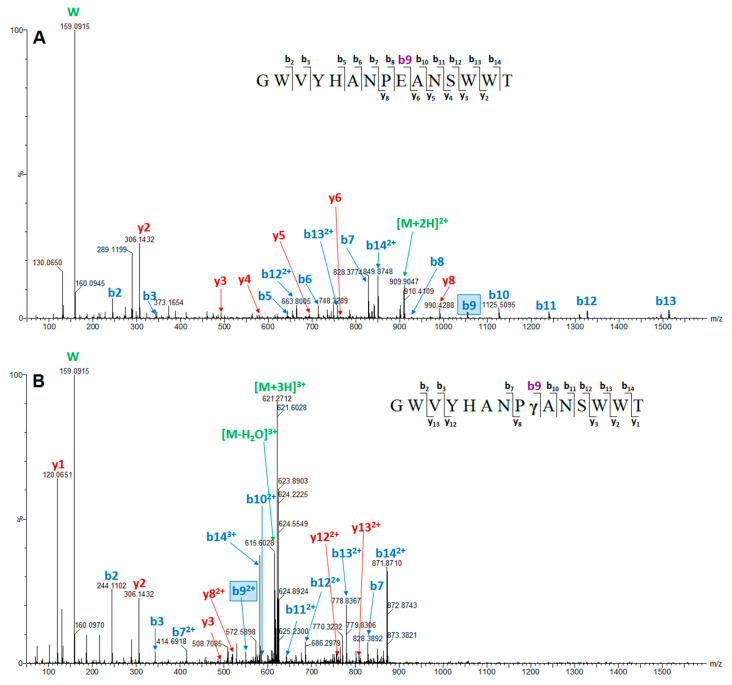
MS/MS spectra of Unmodified (**A**) and Carboxylated [(Gla)9E] (**B**) conomarphin Bt1 or conomarphin Eb2. The diagnostic b9 ions are indicated by a box in the spectra and highlighted in violet in the sequence annotation. The unmodified variant has a b9 ion of 1054.47 Da while the carboxylated variant has a b9 ion of 1098.45 Da.

Figure 5 shows a representative tandem mass spectra of the unmodified conomarphin Bt1 and the carboxylated conomarphin Bt1 [(Gla)9E]. Conomarphin Bt1 [(Gla)9E] is also named conomarphin Eb2 in the ConoServer database [26,27,29]. Meanwhile, conomarphin Bt2 [(Hyp)10P] is also named conomarphin Eb1 in the ConoServer database [26,27,29]. Since Eb1 and Eb2 are both post-translationally modified, we propose a clearer naming scheme (Table 2) that will encapsulate the presence of these PTMs.

Among all the conomarphins identified in this study, we observed that conomarphin Eb1 is highly modified. In addition to the native conomarphin Eb1 peptide, we were able to detect three additional novel sequence variants with singly or doubly modified residues. Conomarphin Eb1 proline at position 10 appears to be always hydroxylated, with additional modifications in position 8 (hydroxyproline) or position 9 (γ-carboxylation of glutamic acid). Incidentally, conomarphin Eb1 found in *C. eburneus* has previously been shown by Mendoza et al. (2019) to have a D-phenylalanine at position 13 [29]. Although its highly likely that the conomarphin Eb1 and variants we detected may also have a D-Phe modification, unfortunately we cannot establish this fact in this study, as these types of modifications are “mass-silent” [4,29,35]. Finally, we also detected conomarphin Bt3, which we propose to be included in ConoServer as conomarphin Eb3. This conomarphin is only predicted to exist based on the precursor peptide from the cDNA transcripts but we show peptide-level evidence of their existence in *C. eburneus*.

To gain insight on the structural changes brought about by differences in peptide backbone or PTMs, we generated 3D structures of the conomarphin variants using the previously solved solution structure of conomarphin (Protein Data Bank ID: 2JQB) as a template [36]. The D-Phe in position 13 in the solution structure was retained in generating the template structures. Four template conomarphin structures were obtained from the four 100-ns MD simulations (Supplementary Figure S1). Each structure corresponds to the possible state of the reference conomarphin at pH levels 3, 5, 7 and 11. The pH levels were selected to follow the protonation and deprotonation of certain residues. Namely, at pH 3, glutamic acid and histidine are protonated. At pH 5, only histidine is charged. Lastly, at pH 11, tyrosine and lysine are deprotonated. Although acidic, pH-dependent deprotonation of the attached functional groups were not applied to the two PTMs, hydroxyproline (O) and carboxylated glutamic acid (γ) and the modified residues were neutral for all pH systems. These sequences were then fitted to the protein backbone of the four template structures. Afterwards, 10-ns MD simulations were performed on each structure. Overall, there were 28 MD systems for the elucidated conomarphins.

Huang et al. showed that the reference conomarphin adopts two unique conformations [36]. At pH 3, the structure resembles a “heart shape” with one flexible arm while the structure is elongated and has a coil-like structure at pH 5. These two conformations were attained in our simulations. Here, the “heart shape” structure present in the reference experimental structure is more pronounced at higher pH.

**Figure 6:**
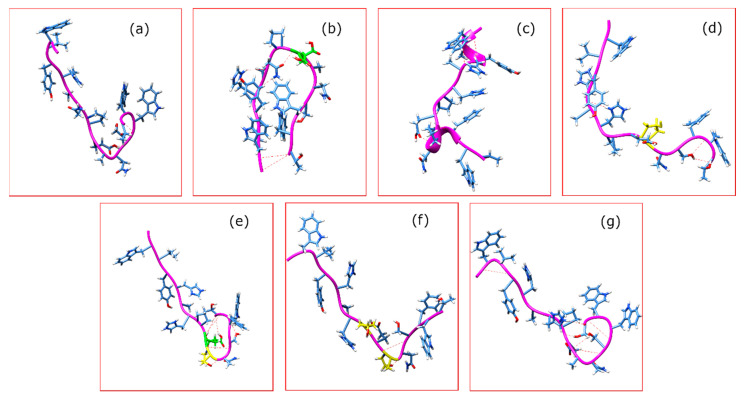
Structure of the seven conomarphin peptides ((**a**): Eb2, (**b**): Eb2[(Gla)9E], (**c**): Eb1, (**d**): Eb1[(Hyp)10P], (**e**): Eb1[(Gla)9E][(Hyp)10P], (**f**): Eb1[(Hyp)8E][(Hyp)10P], and (**g**): Eb3) at pH 3. Residues colored in green are carboxylated glutamic acids while in yellow are the hydroxyprolines. Broken red lines represent the intrapeptide hydrogen bonds.

The predicted structures of the seven conomarphin sequences at pH 3 are presented in Figure 6. There are primarily two conformations for all sequences. The peptides are predicted to adopt either a hairpin-like structure (Figure 6b) or an elongated structure with a twist on the C-terminus end (Figure 6a,c,d). In the latter, the twist seems to be supported by a hydrogen bond network that is facilitated by polar groups. Given their location and polar nature, we surmise that the PTMs help strengthen the said hydrogen bond network. For the hairpin structure, on the other hand, the hydrogen bonds in the previous conformation are replaced by (1) hydrogen bonds between the backbone atoms of the termini and (2) the residues in the middle portion of the peptide. With the exception of conomarphin Eb3, the identity of the termini residues is identical for all peptides. It is apparent that the addition of polar groups (i.e., as in the case of the γ modification) is capable of stabilizing the hydrogen bond networks and, depending on the location of the modifications, would stabilize either conformation.

**Figure 7:**
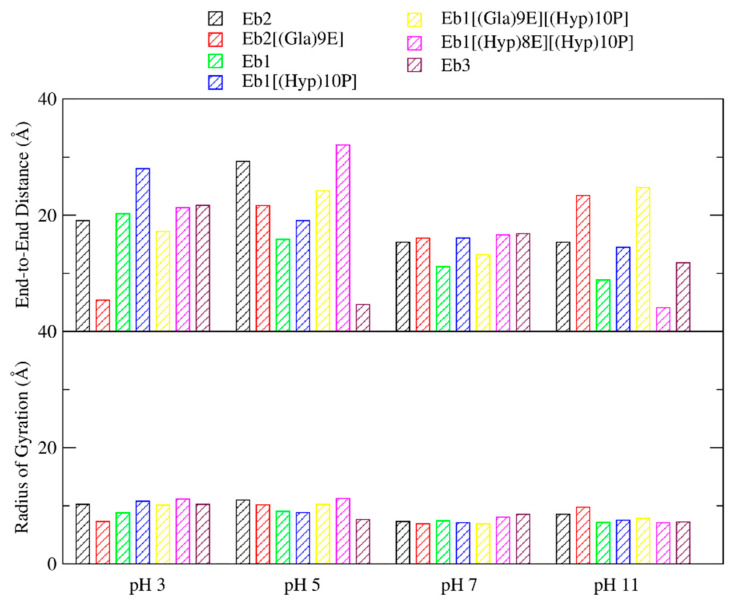
End-to-end distances and radius of gyration (RoG) data of the seven conomarphin peptides at the four pH levels. Calculations were done on the representative structure of each MD system. End-to-end distances were computed as the distance between the alpha carbons of residues 1 and 15. The presented RoG data, on the other hand, are the mass-weighted radius of gyration of all the atoms in the peptides.

We also monitored the overall topology of the peptides by calculating the end-to-end distances and radius of gyration (RoG) values of the predicted structures from the 28 MD systems (Figure 7). For most systems, both the end-to-end distances and RoG values are relatively lower in higher pH conditions. Low end-to-end distances and RoG values imply that the peptides become more compact and spherical at pH 7 and 11. For pH 3 and 5, on the other hand, both values are higher, which suggests that the structures in acidic pH are more elongated and linear.

**Figure 8:**
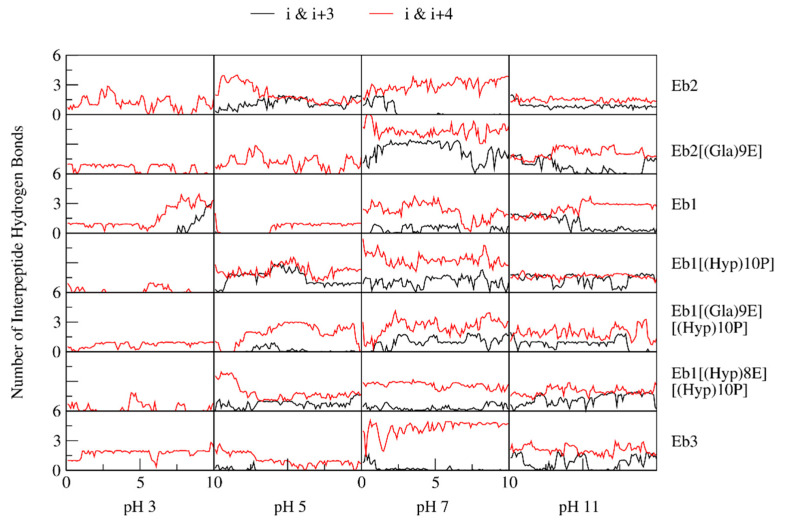
Dynamic intrapeptide hydrogen bonds determined using the geometric criteria. Calculations were done every picosecond and between amino acids of residue distances 3 (*i* & *i*+3) or 4 (*i* & *i*+4). Presented here are the average data for every 0.2 ns time-frame.

These findings are further supported by the calculated intrapeptide hydrogen bonds (Figure 8). The formation of (*i* & *i*+3) and (*i* & *i*+4) hydrogen bonds between amino acid residues imply formation of regular secondary structures 3_10_- and α-helix, respectively. At higher pH, hydrogen-bond formation becomes more prominent. It seems that the change in pH and subsequent deprotonation of glutamic acid and histidine residues allowed the formation of more intrapeptide hydrogen bonds. Combining this information with the results in Figure 7, it can be deduced that the formation of these hydrogen bonds facilitates the shrinking of the peptide, or vice versa.

**Figure 9:**
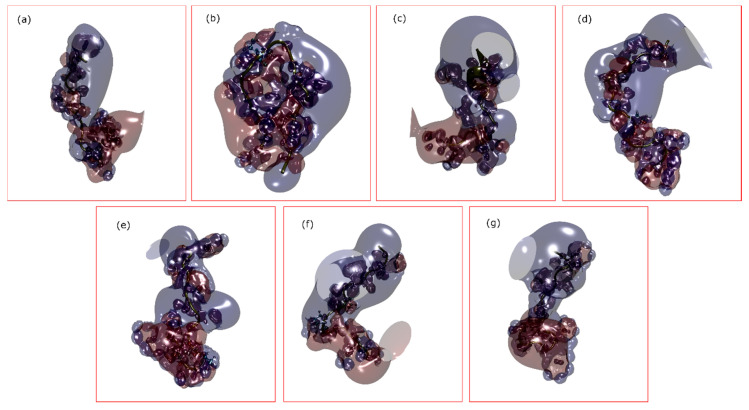
Surface electrostatic potential of the seven conomarphin peptides ((**a**): Eb2, (**b**): Eb2[(Gla)9E], (**c**): Eb1, (**d**): Eb1[(Hyp)10P], (**e**): Eb1[(Gla)9E][(Hyp)10P], (**f**): Eb1[(Hyp)8E][(Hyp)10P], and (**g**): Eb3) at pH 3. Represented using the Corey-Pauling-Koltun (CPK) model are the residues with PTMs. The blue isosurface represents positive potential surfaces while the red isosurface depicts negatively-charged surfaces. Calculations were done using the Adaptive Poisson-Boltzmann Solver (APBS) software. Visualization was done using the VMD software with an isovalue of 1.

We also looked into the possible changes in the electrostatic properties of the conomarphin variants (Figure 9). For the elongated conformation, there is a distinct cluster of negatively-charged regions in the C-terminus. Likewise, there is also formation of a positively-charged region in the N-terminus. The situation is different for the hairpin structure, where there is a larger area of negatively-charged region as compared to the elongated, linear structure.

**Figure 10:**
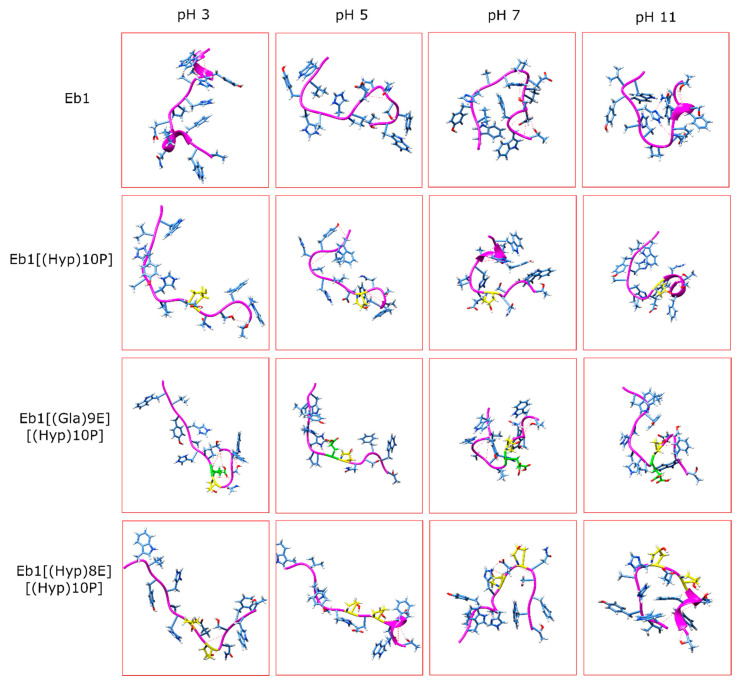
Structures of conomarphin peptides 3–6 (Eb1, Eb1[(Hyp)10P], Eb1[(Gla)9E][(Hyp)10P], and Eb1[(Hyp)8E][(Hyp)10P]) at the four pH levels.

Lastly, we probed the structures of conomarphins at the four pH levels (Figure 10 and Supplementary Figure S2). As shown in Figure 10, the peptides become more compact at higher pH. Interestingly, at pH 7 and 11, a “heart shaped” structure was observed for most of the peptide structures [31]. Indeed, increasing the pH lowers the end-to-end distance and RoG values. Conomarphins Eb1 and Eb1[(Hyp)10P] (Figure 10), for example, shift from the elongated state to a more spherical shape. Conomarphin Eb2, on the other hand, shifts from a hairpin structure to the elongated state (Supplementary Figure S2).

## 3. Discussion

*C. eburneus* is generally regarded as a worm-hunting cone snail that belongs to the Tesseliconus clade [32]. Interestingly, phylogenetic studies have shown that even though *C. eburneus* is a worm-hunter, it is more closely related to fish-hunters, something it has in common with other species in the Tesseliconus clade (*C. tessulatus*, *C. suturatus*, *C. sandwichensis*) [30]. The *C. eburneus* δ-ErVIA has been shown to have striking sequence similarity with δ-TsVIA from *C. tessulatus* [30]. Furthermore, six (6) other peptides identified in this study are also similar to *C. tessulatus* peptides: Ts3-Y01, TsMMSK-021, Ts3.3, Ts3-SGN01, Ts-011, and TsIIIA [26,27].

The diversity of peptides found in *C. eburneus* venom and their similarity to peptides from cone snails with varying prey support the idea that *C. eburneus* may be generalists rather than solely feeding on worms [37]. They may be worm-hunters most of the time but they can attempt to feed on fish or mollusks, probably similar to *C. tessulatus*, which has been previously observed to opportunistically eat dead fish that have been paralyzed by some other fish-hunting cone-snail [29,30].

Alternatively, the existence of multiple, highly abundant conomarphin sequence variants may hint towards the importance of these conopeptides in the defense mechanism of *C. eburneus*. As stated earlier, bioactivity assays of these purified conomarphins revealed that they are not active against fish and mice even at high concentrations. However, they have been found to be active against mollusks even though *C. eburneus* is not known to be a molluscivore [29]. It might be possible that highly abundant conomarphins in the venom duct of *C. eburneus* could function as a defense against other predatory snails.

This study also expanded our knowledge of the diversity of PTMs that can be found in the conomarphin sequences. Unlike the other well-studied conopeptides whose stability, flexibility, and 3D structure are highly dependent on the presence of disulfide bonds, very little is known about the structure of these Cys-free conopeptides, much less the effect of PTMs on the peptide structures. Insights from structural comparisons helped us establish the effects and importance of PTMs on the variants. Overall, the conomarphins appear to adopt two dominant conformations: hairpin-like and elongated. In both structures, the nature of the PTMs helped in the stabilization of hydrogen bond networks at certain conditions. These networks then dictate the structure of these cysteine-free conopeptides. We saw that peptide structures with higher numbers of H-bonds tend to be more spherical. The predicted structures of the conomarphin variants were also found to be sensitive to pH changes. Analogs of Conomarphin Bt-2 (conomarphins Eb1, Eb1[(Hyp)10P], Eb1[(Gla)9E][(Hyp)10P], and Eb1[(Hyp)8E][(Hyp)10P]) have certain structural differences that can be related to the inclusion of PTMs. Summarized in Table 3, we saw that at pH levels 5 and 7, the structure of conomarphin Eb1, a conomarphin without a PTM, can be distinguished from other conomarphins with PTMs.

The computationally-predicted structures presented here might prove to be useful when elucidating the molecular targets of these peptides and the effects of PTMs on the molecular fingerprint of the conomarphins. It appears that the diversification in conomarphin variants (whether due to amino acid substitutions or PTMs) did not necessarily result in significant changes in structure, but the simulations suggest that the presence of particular PTMs may preferentially stabilize certain conomarphin conformations, which in turn may relate back to conomarphin target specificity and selectivity.

## 4. Materials and Methods

### 4.1. Sample Collection and Venom Extraction

*C. eburneus* specimens were collected from the town of Caw-oy in Lapu-Lapu City, Cebu, Philippines. Snails were dissected directly on ice to obtain the venom duct, which was then manually cut into smaller pieces and homogenized. Homogenates were then pooled and centrifuged at 14,000 rpm for 30–45 min at 4 °C. For mass spectrometry analysis, 15 venom ducts were typically pooled together. The resulting supernatant or the crude venom extract (CVE) was collected, dried, and lyophilized for storage.

### 4.2. Peptide Reduction and Alkylation

Dried CVEs from pooled venom ducts were reconstituted in 100 μL of 0.5 M Tris buffer pH 8 (Sigma, St. Louis, MO, USA). The mixture was divided into two: one part was injected directly into the LC-MS while the other half was subjected to reduction/alkylation prior to MS analysis. Peptides were reduced with 10 mM dithiothreitol (Vivantis, Selangor, Malaysia) for 30 min at 65 °C and alkylated using 55–222 mM iodoacetamide (Sigma, St. Louis, MO, USA) for 30 min at room temperature in the dark. The rest of the dried CVE samples were reconstituted in 50 mM of triethylammonium bicarbonate (TEABC, Sigma, St. Louis, MO, USA) buffer pH 8.5 (8.1 μg/μL final peptide concentration) and were reduced and alkylated prior to MS analysis.

### 4.3. Mass Spectrometry

LC-MS/MS analyses were performed using a Xevo-G2-XS-QTOF mass spectrometer (Waters Corporation, Milford, MA, USA) equipped with a Waters Ultra Performance Liquid Chromatography System. Native CVEs and reduced/alkylated extracts were reconstituted in ESI solvent (50:50 acetonitrile with 0.1% formic acid and LC-MS water with 0.1% formic acid) and 3.0 µL aliquots were loaded onto a C18 column (2.1 mm × 50 mm, Acquity, Waters Corporation, Milford, MA, USA) at a flow rate of 0.5 mL/min. Peptides were eluted into the mass spectrometer during a 6.5 min gradient from 5 to 40% B (solvent A, 2:98:0.1 ACN/H2O/formic acid mixture; solvent B, 90:10:0.1 ACN/H2O/formic acid mixture) followed by 1.5 min gradient from 40–95% B. The mass spectrometer was set in positive ion mode and data were acquired with a selected mass range of 400–2000 *m/z*. MS/MS spectra were acquired using data-dependent acquisition (DDA) mode selecting ions with up to +6 charge state. Peak lists from ESI-MS and MS/MS spectra were generated using MassLynx V4.1 (Waters Corporation, Milford, MA, USA).

Complementary LC-MS/MS analyses were performed on a TripleTOF 5600 system coupled to a nanoACQUITY UPLC (AB SCIEX Concord, ON, Canada) and a LTQ Orbitrap Velos (Thermo Electron, Bremen, Germany). Native CVEs and reduced/alkylated extracts were reconstituted in 0.1% formic acid to a final concentration of 0.25 μg/μL and 2.0 μL aliquots were loaded onto a C18-aqueous (AQ) column (Waters Corporation, Milford, MA, USA). Elution was carried out using a linear gradient of 2–35% solvent at a flow rate of 0.5 mL/min. Likewise, the mass spectrometer was set in positive ion mode and data were acquired with a selected mass range of 300–1600 *m/z*. MS/MS spectra were acquired using the data-dependent acquisition (DDA) mode, selecting ions with a +2 to +4 charge state. Venom peptide samples for the Orbitrap were loaded into a C18 bridged ethylsiloxane/silica hybrid (BEH) column (75 μm ID, 25 cm length, Waters Corporation, Milford, MA, USA) packed with 1.7 μm particles with a pore width of 130 Å. A segmented gradient elution from 5% to 35% solvent B was carried out for 90 min at a flow rate of 300 nL/min and a column temperature of 35 °C. The mass spectrometer was set in positive ion mode and data were acquired with a selected mass range of 350–1600 *m/z*. MS/MS spectra were acquired using the data-dependent acquisition (DDA) mode, selecting ions with +2 or higher charge states. Peak lists from the TripleTOF and Orbitrap were generated and exported to mgf format by Mascot Distiller v2.3.2 (Matrix Science, London, UK).

### 4.4. Mass Spectrometric Data Search and Analysis

Conopeptide identification was performed using the ConoMass tool in ConoServer against the ConoServer database with 6275 peptide entries (www.conoserver.org) and against a user-generated database derived from the *C. eburneus* transcriptome library (149 transcript entries) [26,27,29]. Intact precursor masses were matched within a 10 ppm error and included the following post-translational modifications (PTMs): amidation of C-terminus, hydroxylation of proline and valine, pyroglutamylation of N-terminus glutamine, γ-carboxylation of glutamic acid, bromination of tryptophan, and sulfation of tyrosine. Comparison of native and reduced/alkylated CVEs allowed the identification of the number of disulfide bonds present in peptides. The number of disulfide bonds present in conopeptides was deduced from the observed incremental mass shifts upon alkylation of the cysteine residues. Sequence verification by MS/MS was performed by peptide–spectrum matching using in-house programs as well as Proteome Discoverer (Thermo Fisher Scientific, Waltham, MA, USA). Precursor mass tolerance was set to 20 ppm while fragment mass tolerance was set to ±0.1 Da. The following PTMs were selected in Proteome Discoverer: cysteine carbamidomethylation, methionine oxidation, lysine acetylation, tyrosine sulfation, γ-carboxylation of glutamic acid, and amidation of C-terminus. A q-value cut-off of 0.01 was set to ensure the confidence of the assigned peptide–spectrum matches. Finally, candidate sequences were reported if the sequence coverage was >45% and >70% of the expected bond cleavages were detected.

### 4.5. In Silico MD Simulation

A solution structure of conomarphin (PDB ID: 2JQB) was used as a template for the succeeding MD simulations [36]. Previously, the structure of conomarphins has been observed to be highly pH dependent. As such, we speculate that this pH-dependent structure would also be true for conomarphin variants given their cysteine-free nature.

To obtain initial conformations for the MD studies, the reference conomarphin was subjected to four 100-ns MD simulations. Each simulation corresponds to a different pH level: pH 3, 5, 7 and 11. Protonation of the reference conomarphin was done using the H++ web server [38,39,40]. Then, these structures were solvated with TIP3P water molecules in a cubic box [41]. TIP3P water molecules were added to the system until the distance between the conomarphin atoms and the edge of the box was at least 10 Å. An appropriate number of K^+^ or Cl^−^ ions was added to neutralize the charge of the system. In this study, the ff14SB AMBER force field was used to describe the protein [42].

The MD simulations were performed using the sander program of AmberTools19 [43]. Before performing the production run, a five-step minimization procedure was followed. First, 2000 minimization steps were done on the water molecules and the counterions with the position of the peptide restrained. Next, the non-peptide atoms underwent 5000 steps of NPT equilibration. Afterwards, all atoms were minimized using the same protocols as the first step. Fourth, the water molecules and counterions were heated from 0 K to 300 K in an NVT ensemble. Here, the position of peptide atoms was also restrained. Lastly, non-peptide atoms were subjected to 500 picoseconds of NPT equilibration. After minimization, the systems then went on 100-ns NPT production runs.

Both sections of the MD simulations (minimization and production) used the SHAKE constraints for all bonds involving hydrogen [44]. Non-bonded interactions used a cutoff of 10 Å. For steps that made use of position restraints, a force constant of 10 Å was applied. Minimization steps 2 and 4 used the Berendsen temperature coupling scheme to regulate the temperature [45]. Minimization step 5 and production runs, on the other hand, used the Langevin thermostat, with a collision frequency of 2 ps^−1^ to maintain the temperature [46]. These two steps also used isotropic position scaling and a relaxation time of 1 ps to control the pressure. For the production runs, a time step of 2 fs was used.

After the MD simulations, the last frame of each simulation was extracted and used as the initial conformation of the succeeding MD simulations of the seven conomarphin sequences. A total of 28 (4 pH levels × 7 conomarphin sequences) MD systems were prepared. These systems followed the same procedure as the reference conomarphin for the preparation step. For the PTMs, the hydroxyproline residues used the HYP force field available in ff14SB. Carboxylated glutamic acid residues, on the other hand, used the CGU force field of the Forcefield_PTM force fields [47,48]. These two residues, however, were maintained at neutral state for all MD simulations. There are no currently available force fields for the deprotonated states of these two PTMs.

Except for minimization step 3 and production, the 28 MD systems followed the same MD protocols as the reference conomarphin. For minimization step 3, the number of steps was increased to 10^4^. For production runs, the length of the simulations was limited to 10 ns.

After the MD simulations, each system underwent biophysical analyses using the AmberTools program cpptraj, and in-house scripts used in a previous study [49,50]. To determine if the protein is elongated, end-to-end distance of the representative structures were obtained. This corresponds to the distance between the α carbons of residues 1 and 15. These results were correlated with the radius of gyration results to obtain an overall insight on the structure of the conomarphin sequences.

To determine possible formation of secondary structures, internal hydrogen bonds were quantified. Determination of H-bond formation was done using the geometric criterion (GC). Here, an H-bond between carboxyl oxygen and the nitrogen of the amine group is assigned if the corresponding distance, d, and angle, θ, fit the following criteria: d ≤ 3.5 Å and 120° ≤ θ ≥ 180°. Calculations were done between residues separated by 3 (*i* & *i* + 3) and 4 (*i* & *i* + 4) residues [51].

Electrostatic attributes (electrostatic isosurfaces) of the structures were calculated using the Adaptive Poisson-Boltzmann Solver (APBS) web server. Visualization of results were performed using the Visual Molecular Dynamics (VMD) and UCSF Chimera software [52,53].

## Figures and Tables

**Table 1:** Conopeptides identified in this study through tandem mass spectrometry.

Peptide Name (in *C. eburneus* venom duCt transCriptome) ^a^	Peptide Name (in ConoServer Database)	ConoServer ID	Peptide SequenCe ^†^	Gene Super-Family	Cysteine Frame-Work	Number of Disulfide Bonds
CE102	ErVIA	P06773	**C**AGIGSF**C**GLPGLVD**CC**SGR**C**FIV**C**LP	O1	VI/VII	3
CE030		*	TALEDADMKTEKGVLSGIMSNLGTVGNMVGGF**CC**TVYSG**CC**AE	T	V	2
CE031		*	AALEDADMKTAKGILSNIMGNLGNIGNMAGSF**CC**SVYSG**CC**PE	T	V	2
CE103		*	FLGLIGPITSIAGKL**CC**TVSVSF**CC**NE	T	V	2
CE120		*	TLQRHWAKFL**CC**PEDDW**CC**	T	V	2
CE123		*	DL**C**PH**C**PNG**C**HVDRT**C**I	L	XIV	2
CE133		*	L**C**PPM**C**RS**C**SN**C**	L	XIV	2
CE133 **[(MOx)5M]**		*	L**C**PP(MOx)**C**RS**C**SN**C**	L	XIV	2
	Conomarphin-Bt1	P05978	GWVYHANPEANSWWT	M	Not assigned	0
	Conomarphin-Eb2	P08992	GWVYHANP(Gla)ANSWWT	M	Not assigned	0
	Conomarphin-Bt2	P05979	GWVYHAHPEPNSFWT	M	Not assigned	0
	Conomarphin-Eb1	P08991	GWVYHAHPEONSFWT	M	Not assigned	0
	Conomarphin-Bt2 [(Gla)9E][(Hyp)10E]	P05979	GWVYHAHP(Gla)ONSFWT	M	Not assigned	0
	Conomarphin-Bt2 [(Hyp)9E] [(Hyp)10E]	P05979	GWVYHAHOEONSFWT	M	Not assigned	0
CE019	Conomarphin-Bt3	P05980	GWVYHAHPDANSWWS	M	Not assigned	0
CE138	Contryphan-Bt1 [(Hyp)3E]	P05977	G**C**OPGLW**C**(Nh2)	O2	Not assigned	1
	Eu3.5	P04637	**CC**VV**C**NAG**C**SGN**CC**P	M	III	3
CE135	Ts-011	P02712	G**CC**EDKT**CC**FI	T	V	2
CE128	Ts3.3	P03167	**CC**SRY**C**YI**C**IP**CC**PN	M	III	3
	Ts3-SGN01	P05089	**CC**VV**C**NAG**C**SGN**CC**S	M	III	3
CE119	TsIIIA	P07525	G**CC**RWP**C**PSR**C**GMAR**CC**SS	M	III	3
	TsMMSK-021	P03154	**CC**DWP**C**TIG**C**VP**CC**LP	M	III	3
	TsVIA	P06849	**C**AAFGSF**C**GLPGLVD**CC**SGR**C**FIV**C**LL	Unknown	VI/VII	3
CE124	Ts3-Y01	P04949	R**CC**ISPA**C**NDT**C**Y**CC**QD	M	III	3

^a^ Peptides predicted from the transcriptome are named with the identifier CE followed by a number (e.g., CE001). ^†^ Cysteine residues are highlighted in red; * Found in the transcriptome but not in the ConoServer database; (Nh2)—N terminus amidation; O—proline hydroxylation; (Gla)—glutamic acid carboxylation; (MOx)—methionine oxidation.

**Table 2:** *C. eburneus* conomarphin variants identified in this study through tandem mass spectrometry (MS/MS).

Conomarphin Number	MS/MS Verified Sequence	PTMs Identified by MS	Conomarphin Name in ConoServer Database	Proposed Conomarphin Name ^a^
**1**	GWVYHANPEANSWWT^c^	none	Bt1	Eb2
**2**	GWVYHANPγANSWWT	γ-carboxylation (E)	Eb2	Eb2[(Gla)9E]
**3**	GWVYHAHPEPNSFWT^c^	none	Bt2	Eb1
**4**	GWVYHAHPEONSFWT	Hydroxylation (P)	Eb1 ^b^	Eb1[(Hyp)10P]
**5**	GWVYHAHPγONSFWT ^d^	γ-carboxylation (E); Hydroxylation (P)	None	Eb1[(Gla)9E][(Hyp)10P]
**6**	GWVYHAHOEONSFWT^d^	Hydroxylation (P) x 2	None	Eb1[(Hyp)8E][(Hyp)10P]
**7**	GWVYHAHPDANSWWS^c^	none	Bt3	Eb3

Highlighted in yellow and written in boldface are residues that are different in one or more peptides. Conomarphins 2, 4, 5 and 6 have PTMs. An underlined F indicates a D-Phe residue. ^a^ Proposed name for new *C. eburneus* conomarphins reported in this study; ^b^ Conomarphin Eb1 has D-Phe at position 13; ^c^ this study provides peptide-level evidence; ^d^ novel conomarphin variants.

**Table 3:** Summary of the biophysical properties of select conomarphins at different pH levels.

pH Levels	Conomarphin	Properties		Possible Effect of PTMs
		Ave. # of H-Bonds	End-to-End Distance (Å)	
**pH 3**	Eb1	1.9673	20.2473	There are no significant differences in the properties.
	Eb1[(Hyp)10P]	0.1724	28.0155	
	Eb1[(Gla)9E][(Hyp)10P]	0.7245	17.2082	
	Eb1[(Hyp)8E][(Hyp)10P]	0.2952	21.3046	
**pH 5**	Eb1	0.5768	15.8414	Conomarphins with PTMs are more elongated and polarized.
	Eb1[(Hyp)10P]	3.6198	19.0677	
	Eb1[(Gla)9E][(Hyp)10P]	2.0044	24.1722	
	Eb1[(Hyp)8E][(Hyp)10P]	2.586	32.0786	
**pH 7**	Eb1	2.5545	11.1528	Conomarphins without PTM are more spherical.
	Eb1[(Hyp)10P]	4.4091	16.0787	
	Eb1[(Gla)9E][(Hyp)10P]	3.5531	13.2177	
	Eb1[(Hyp)8E][(Hyp)10P]	2.9578	16.6192	
**pH 11**	Eb1	3.3795	8.8539	The properties are too variable to derive a conclusion.
	Eb1[(Hyp)10P]	2.9953	14.4752	
	Eb1[(Gla)9E][(Hyp)10P]	2.6705	24.7668	
	Eb1[(Hyp)8E][(Hyp)10P]	3.1841	4.083	

## Supplementary Materials

The following are available online at https://www.mdpi.com/1660-3397/18/10/503/s1, Figure S1: Representative structures of the reference conomarphin at pH levels (a) 3, (b) 5, (c) 7, and (d) 11, Figure S2: Structures of conomarphin peptides 1,2, and 7 (Eb2, Eb2[(Gla)9E], and Eb3) at the four pH levels. Table S1: Conopeptides identified in the *C. eburneus* venom duct.
