# Supplementary material for: Identification of Conomarphin Variants in the Conus eburneus Venom and the Effect of Sequence and PTM Variations on Conomarphin Conformations
Source: Mar Drugs. 2020 Oct 1;18(10):503. doi: 10.3390/md18100503 (PMC7601563; doi:10.3390/md18100503)
Supplement: Supplementary file 1 [file marinedrugs-18-00503-s001.pdf]

marine drugs

MDPI

Article

Identification of conomarphin variants in the *Conus eburneus* venom and the effect of sequence and PTM variations on conomarphin conformations [Supplementary Materials]

Corazon Ericka Mae M. Itang [1,2], Jokent T. Gaza [1], Dan Jethro M. Masacupan [2], Dessa Camille R. Batoctoy [1], Yu-Ju Chen [3], Ricky B. Nellas [1] and Eizadora T. Yu [1,2,*]

[1] Institute of Chemistry, College of Science, University of the Philippines, Diliman, Quezon City, Philippines 1101; erickaitang@gmail.com (C.E.M.M.I.); jtgaza@up.edu.ph (J.T.G.); drbatoctoy@up.edu.ph (D.C.R.B.); rbnellas@up.edu.ph (R.B.N.)
[2] Marine Science Institute, College of Science, University of the Philippines, Diliman, Quezon City, Philippines 1101; djmasacupan@gmail.com
[3] Institute of Chemistry, Academia Sinica, No. 128, Section 2, Academia Rd, Nankang, Taipei 115, Taiwan; yujuchen@gate.sinica.edu.tw.
* Correspondence: etyu@up.edu.ph; Tel.: +632-922-3959

Figure S1. Representative structures of the reference conomarphin at pH levels (a) 3, (b) 5, (c) 7, and (d) 11. These structures are the last frames of the four MD simulations. Intrapeptide hydrogen bonds are represented by broken red lines. The position of PRO8 at each structure is pointed by a red arrow.

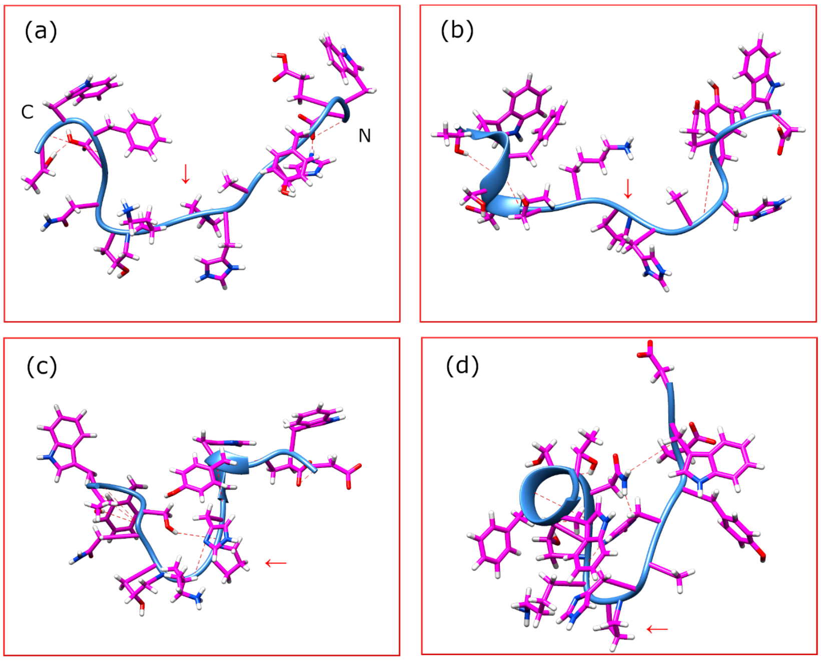

Figure S2. Structures of conomarphin peptides 1,2, and 7 (Eb2, Eb2[(Gla)9E], and Eb3) at the four pH levels.

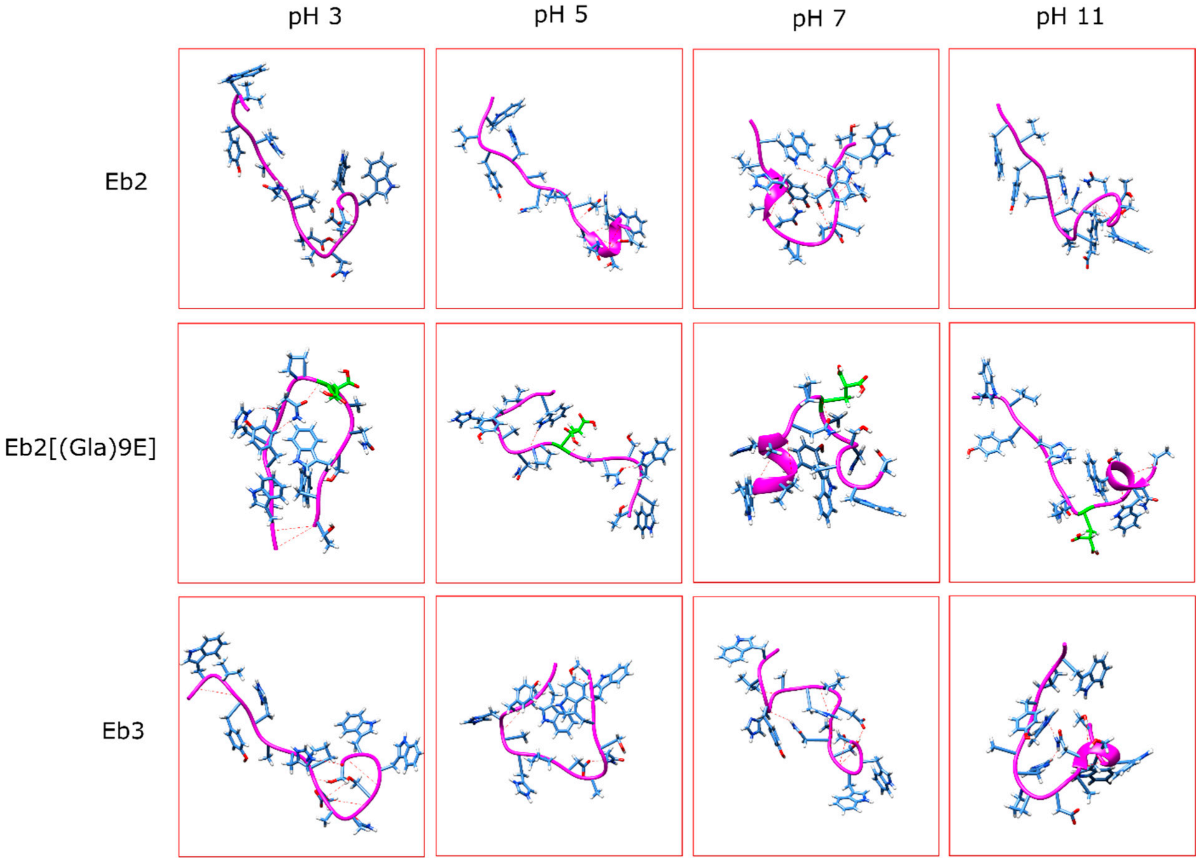

Table S1. Conopeptides identified in the *C. eburneus* venom duct.

Peptide Name (in *C. eburneus* venom duct transcriptome) †	Peptide Name (in ConoServer database)	ConoServer ID	Peptide Sequence ‡	Gene superfamily	Cysteine Framework	Number of disulfide bonds	MS2-verified?
CE102	ErVIA	P06773	CAGIGSFCGLPGLVDCCSGRCFIVCLP	O1	VI/VII	3	Y
CE030		*	TALEDADMKTEKGVLSGIMSNLGTVGNMVGGFCCTVYSGCCAE	T	V	2	Y
CE031		*	AALEDADMKTAKGILSNIMGNLGNIGNMAGSFCCSVYSGCCPE	T	V	2	Y
CE103		*	FLGLIGPITSIAGKLCCTVSVSFCCNE	T	V	2	Y
CE120		*	TLQRHWAKFLCCPEDDWCC	T	V	2	Y
CE123		*	DLCPHCPNGCHVDRTCI	L	XIV	2	Y
CE133		*	LCPPMCRSCSNC	L	XIV	2	Y
CE133 [(MOx)5M]		*	LCPP(MOx)CRSCSNC	L	XIV	2	Y
	Conomarphin-Bt1	P05978	GWVYHANPEANSWWT	M	Not assigned	0	Y
	Conomarphin-Eb2	P08992	GWVYHANP(Gla)ANSWWT	M	Not assigned	0	Y
	Conomarphin-Bt2	P05979	GWVYHAHPEPNSFWT	M	Not assigned	0	Y
	Conomarphin-Eb1	P08991	GWVYHAHPEONSFWT	M	Not assigned	0	Y
	Conomarphin-Bt2 [(Gla)9E][(Hyp) 10E]	P05979	GWVYHAHP(Gla)ONSFWT	M	Not assigned	0	Y
	Conomarphin-Bt2 [(Hyp)9E] [(Hyp)10E]	P05979	GWVYHAHOEONSFWT	M	Not assigned	0	Y
CE019	Conomarphin-Bt3	P05980	GWVYHAHPDANSWWS	M	Not assigned	0	Y
CE138	Contryphan-Bt1 [(Hyp)3E]	P05977	GCOPGLWC(Nh2)	O2	Not assigned	1	Y
	Eu3.5	P04637	CCVVCNAGCSGNCCP	M	III	3	Y
CE135	Ts-011	P02712	GCCEDKTCCFI	T	V	2	Y

CE128	Ts3.3	P03167	CCSRYCYICIPCCPN	M	III	3	Y
	Ts3-SGN01	P05089	CCVVCNAGCSGNCCS	M	III	3	Y
CE119	TsIIIA	P07525	GCCRWPCPSRCGMARCCSS	M	III	3	Y
	TsMMSK-021	P03154	CCDWPCTIGCVPCCLP	M	III	3	Y
	TsVIA	P06849	CAAFGSFCGLPGLVDCCSGRCFIVCLL	Unknown	VI/VII	3	Y
CE124	Ts3-Y01	P04949	RCCISPACNDTCYCCQD	M	III	3	Y
CE026		*	QFDC(hVa)DGGEQCR(hVa)NSNCCS(sTy)LCCOKSIGKRCAIQRS VGCDDFRI	I1	XI	4	N
CE035		*	(Gla)CQQHANCDSGKWCCEECCASSNCGCKGEDISGGVIVQVC DC	N	?	5	N
CE051		*	CTDDSQFCNPSSHNCCSGTCIDEGGSGVCAIVPVTV	O1	VI/VII	3	N
CE096		*	DCTOOGGACGGHAHCCSKSCNIMASTCQ	O1	VI/VII	3	N
CE098		*	VSCGDTCINSDECPSSCNTCLHALCKS	P	IX	3	N
CE115		*	CPWCGSTCCPPHYCQGVTCVYV	O1	VI/VII	3	N
CE121		*	GG(Gla)PRV(Gla)ASR(Gla)RLQ(Gla)IGR(Nh2)	conantokin	None	0	N
	Eu3.3	P04639	CCQAACSOWLCLOCC(Nh2)	M	III	3	N
CE128	Ts3.3	P03167	CCSRYCYICIOCCPN	M	III	3	N
	Ts3.5	P03171	DCCVMPWCDGACDCCVSS(Nh2)	M	III	3	N
	Ts5.1	P03187	TLQRHWAKSLCCO(Gla)DAWCCSHD(Gla)(Nh2)	T	V	2	N
	Ts6.6	P03246	GD(Gla)(Gla)CN(Gla)(sTy)CDDRNK(Gla)CCGRTNGHORCAN(hV a)CF(Nh2)	O3	VI/VII	3	N

† Peptides having names starting with CE were predicted from the transcripts
‡ Cysteine residues are highlighted in red
* Found in the transcriptome but not in the ConoServer database
(Nh2) - N terminus amidation; O - proline hydroxylation; (hVa) - valine hydroxylation; (Gla) - glutamic acid carboxylation; (sTy) - tyrosine sulfation; (MOx) - methionine oxidation
